# Effects of the COVID-19 pandemic on acute coronary syndromes in Germany during the first wave: the COVID-19 collateral damage study

**DOI:** 10.1007/s00392-022-02082-3

**Published:** 2022-08-17

**Authors:** Uwe Zeymer, Vusal Ahmadli, Steffen Schneider, Karl Werdan, Michael Weber, Sven Hohenstein, Gerhard Hindricks, Steffen Desch, Andreas Bollmann, Holger Thiele

**Affiliations:** 1 Medizinische Klinik B, Klinikum Ludwigshafen, Klinik für Innere Medizin/Kardiologie, Bremserstr. 79, 67063 Ludwigshafen, Germany; 2 Institut für Herzinfarktforschung, Ludwigshafen, Germany; 3 DGK Zentrum für Kardiologische Versorgungsforschung, Düsseldorf, Germany; 4 Department of Cardiology, Heart Center Leipzig at University of Leipzig, Strümpellstraße 39, 04289 Leipzig, Germany; 5 Leipzig Heart Institute, Leipzig, Germany; 6 Universität Halle, Halle, Germany; 7 Verband der Leitenden Krankenhausärzte Deutschlands e.V., Karlsruhe, Germany; 8 Department of Electrophysiology, Heart Center Leipzig at University of Leipzig, Leipzig, Germany

**Keywords:** Acute myocardial infarction, COVID-19 pandemic, Mortality, Revascularization, Collateral damage

## Abstract

**Background:**

Reports about the influence of the COVID-19 pandemic on the number of hospital admissions and in-hospital mortality during the first wave between March and May 2020 showed conflicting results and are limited by single-center or limited regional multicenter datasets. Aim of this analysis covering all German federal states was the comprehensive description of hospital admissions and in-hospital mortality during the first wave of the COVID-19 pandemic.

**Methods and results:**

We conducted an observational study on hospital routine data (§21 KHEntgG) and included patients with the main diagnosis of acute myocardial infarction (ICD 21 and ICD 22). A total of 159 hospitals included 36,329 patients in the database, with 12,497 patients admitted with ST-elevation myocardial infarction (STEMI) and 23,832 admitted with non-ST-elevation myocardial infarction (NSTEMI). There was a significant reduction in the number of patients admitted with STEMI (3748 in 2020, 4263 in 2019 and 4486 in 2018; *p* < 0.01) and NSTEMI (6957 in 2020, 8437 in 2019 and 8438 in 2020; *p* < 0.01). These reductions were different between the Federal states of Germany. Percutaneous coronary intervention was performed more often in 2020 than in 2019 (odds ratio 1.13, 95% confidence interval [CI] 1.06–1.21) and 2018 (odds ratio 1.20, 95% CI 1.12–1.29) in NSTEMI and more often than in 2018 (odds ratio 1.26, 95% CI 1.10–1.43) in STEMI. The in-hospital mortality did not differ between the years for STEMI and NSTEMI, respectively.

**Conclusions:**

In this large representative sample size of hospitals in Germany, we observed significantly fewer admissions for NSTEMI and STEMI during the first COVID-19 wave, while quality of in-hospital care and in-hospital mortality were not affected.

**Graphical abstract:**

Admissions for STEMI and NSTEMI during the months March to May over 3 years and corresponding in-hospital mortality for patients with STEMI and NSTEMI in 159 German hospitals. (*p*-value for admissions 2020 versus 2019 and 2018: < 0.01; *p*-value for mortality: n.s.)

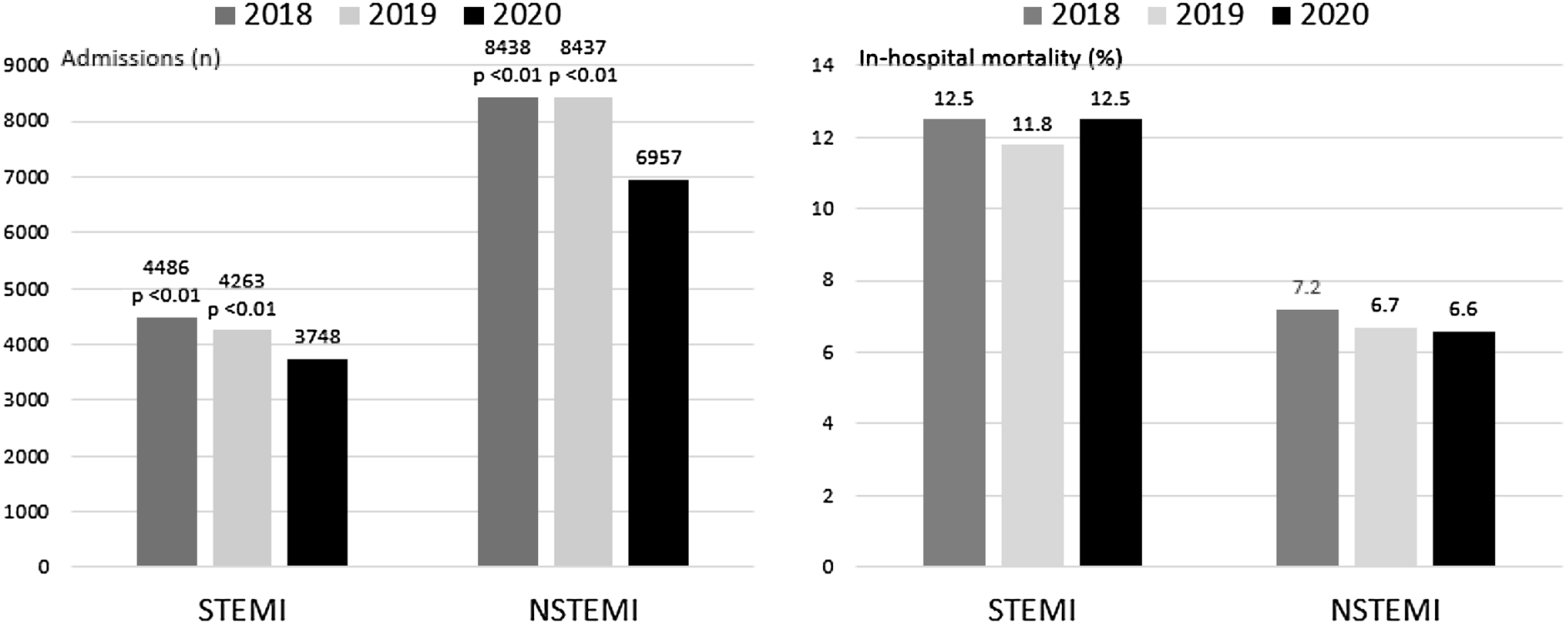

## Introduction

The outbreak of the COVID-19 pandemic was reported in December 2019 in Wuhan, China [1] and recognized as a worldwide pandemic on March 11, 2020. The Severe Acute Respiratory Syndrome Corona Virus 2 (SARS-CoV-2) leads to pneumonia in around 10% of patients, which is severe in around 2%. The outbreak of the pandemic in Germany in spring 2020 led to changes in the health system, with the aim of being able to care for the expected increasing number of seriously ill patients with SARS-CoV-2 [2]. During the COVID-19 pandemic, reduced hospitalization rates for various cardiovascular and non-cardiovascular diseases have been observed in multiple registries [3–16]. Numerous studies on this topic are also available from Germany [17–21]. The majority of these observational data are limited by single-center or limited regional multicenter designs. While some studies showed reductions in the number of admissions for both STEMI and NSTEMI and an increase in acute coronary syndrome (ACS) mortality others report only an effect on admissions for non-ST-elevation myocardial infarction (NSTEMI) with no effect on mortality and morbidity [22, 23].

Since these conflicting data might be possibly due to the limited number of participating hospitals and patients, the current “COVID-19 Collateral Damage” analysis thought to collect the data from a large number of various centers across Germany. Major aim was to determine the influence of the COVID-19 pandemic in the first wave (from March 2020 to May 2020) compared to the corresponding periods in 2018 and 2019 on the hospitalization rates of patients with acute myocardial infarction (including STEMI and NSTEMI) and the respective in-hospital mortality in a large nationwide analysis.

## Methods

For this analysis routine data (§21 KHEntgG) of German hospitals were centrally collected and analyzed. Inclusion criteria were admissions with ICD (International Statistical Classification of Diseases and Related Health Problems ICD-10-GM) code I.21 (acute myocardial infarction) and I.22 (acute recurrent myocardial infarction). Patients admitted for other reasons and diagnosed with acute myocardial infarction during their hospital stay were excluded. The dataset included year and month of birth, sex, admission date, admission diagnosis, discharge date, discharge mode, ICD-codes, OPS codes and OPS dates, intensive care duration, duration of mechanical ventilation and in-hospital mortality.

### Participating hospitals

**Fig. 1:**
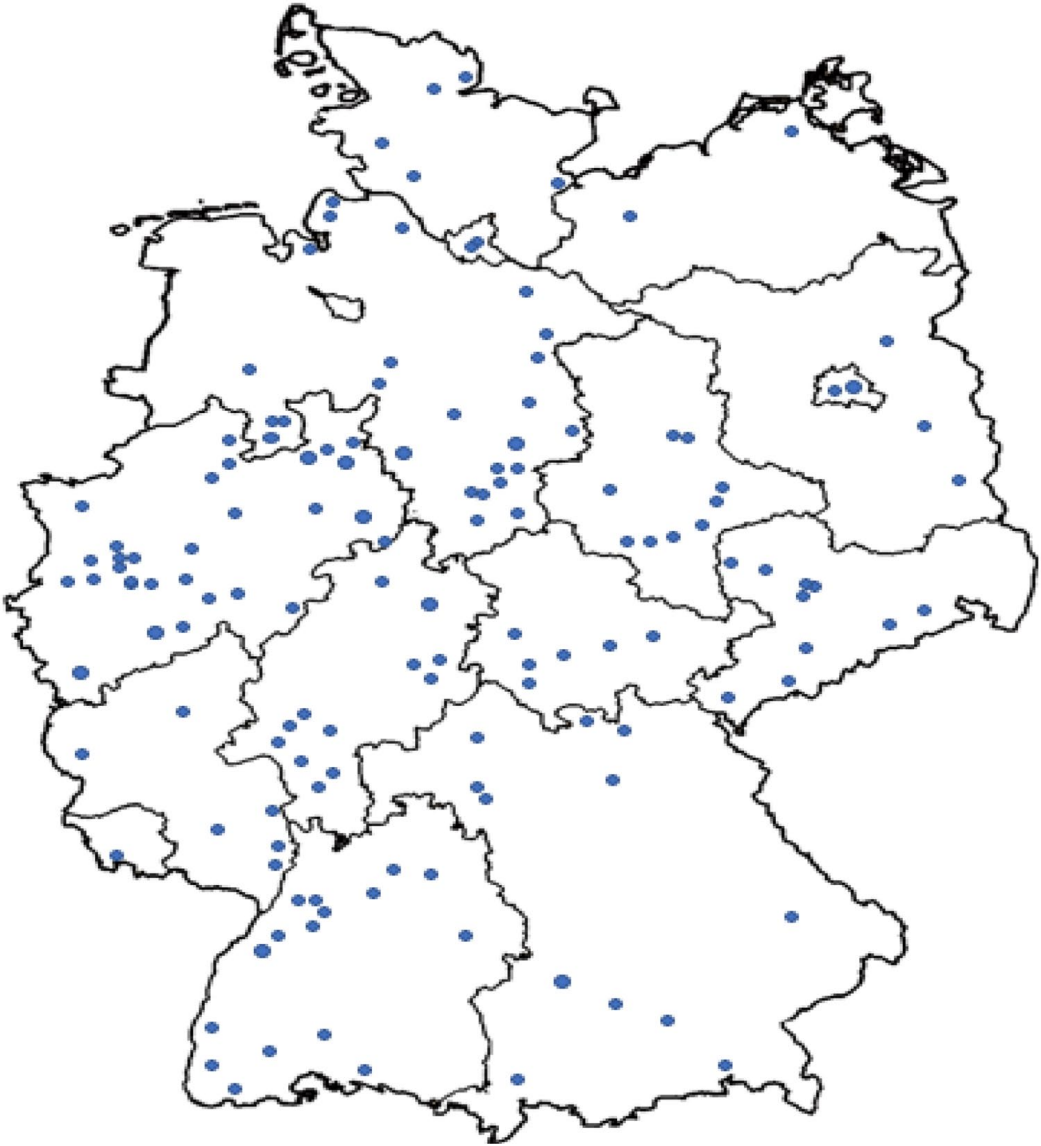
Distribution of participating hospitals in Germany

A total of 221 hospitals with the capability to care for patients with acute myocardial infarction were invited to participate in the COVID-19 Collateral Damage Study. Of these, 159 provided their data. The map with participating hospitals shows the distribution within Germany (Fig. 1).

### Study periods

The first official COVID-19 patient in Germany was reported on January 27, 2020. On March 13 and 17, the federal and state governments decided on a series of measures to prepare the health system for the expected increase in the number of COVID-19 cases. This included the postponement of elective surgeries (e.g., coronary artery bypass graft operations) and elective invasive procedures (e.g., coronary angiographies and percutaneous coronary interventions [PCI]) and an emergency plan for hospitals, with which the intensive care capacities could be expanded. At the same time, quarantine measures for travelers from abroad and travel restrictions were decided. On March 22, 2020, the federal government and the states agreed on a comprehensive “restriction of social contacts” [22].

In June, a decline in COVID-19 cases in Germany was reported and restrictions were eased. Therefore, we have selected the period between March and May 2020 and compared it to the respective periods in the years 2018 and 2019.

### Statistical analysis

The data were provided directly by the centers or extracted from QlikView (QlikTech, Radnor, Pennsylvania, USA). The distribution of patient characteristics, comorbidities, procedures and in-hospital outcome is described using absolute numbers and percentages for the years 2018, 2019, and 2020, separately for STEMI and NSTEMI. Only for patients with complete information, weighted Elixhauser comorbidity index is calculated using AHRQ algorithm [25] and the distribution is given by mean and standard deviation. Regarding baseline characteristics, comparisons between years of admission are performed using Chi-Square or Kruskal–Wallis test.

Regarding procedures and outcome, comparisons between years of admission are based on generalized linear mixed models (GLMM) with logit link function with hospitals as a random factor [23]. For numbers of admission, Poisson GLMMs is used. The impact was estimated with the lme4 package (version 1.1–26) in the R environment for statistical computing (version 4.0.2) (R Core Team 2020) [24]. Odds ratios (OR) and incidence rate ratios (IRRs) (calculated by exponentiation of the regression coefficients) along with 95% confidence intervals (CI) (for the comparisons of the two periods) are reported.

In addition, the influence of the volume of SARS-CoV-2 infections in the different German federal states on the change in numbers of admission STEMI/NSTEMI was analyzed via multivariate mixed Poisson regression. In the model, tertiles of federal states regarding cumulative summed SARS-CoV-2 volume between January and May 2020 per 100.000 inhabitants and the year of admission are used as independent parameters.

## Results

### Admissions and patient characteristics

A total of 159 hospitals (Fig. 1) included 36,329 patients in the database, with 12,497 patients admitted with STEMI and 23,832 admitted with NSTEMI.

**Fig. 2:**
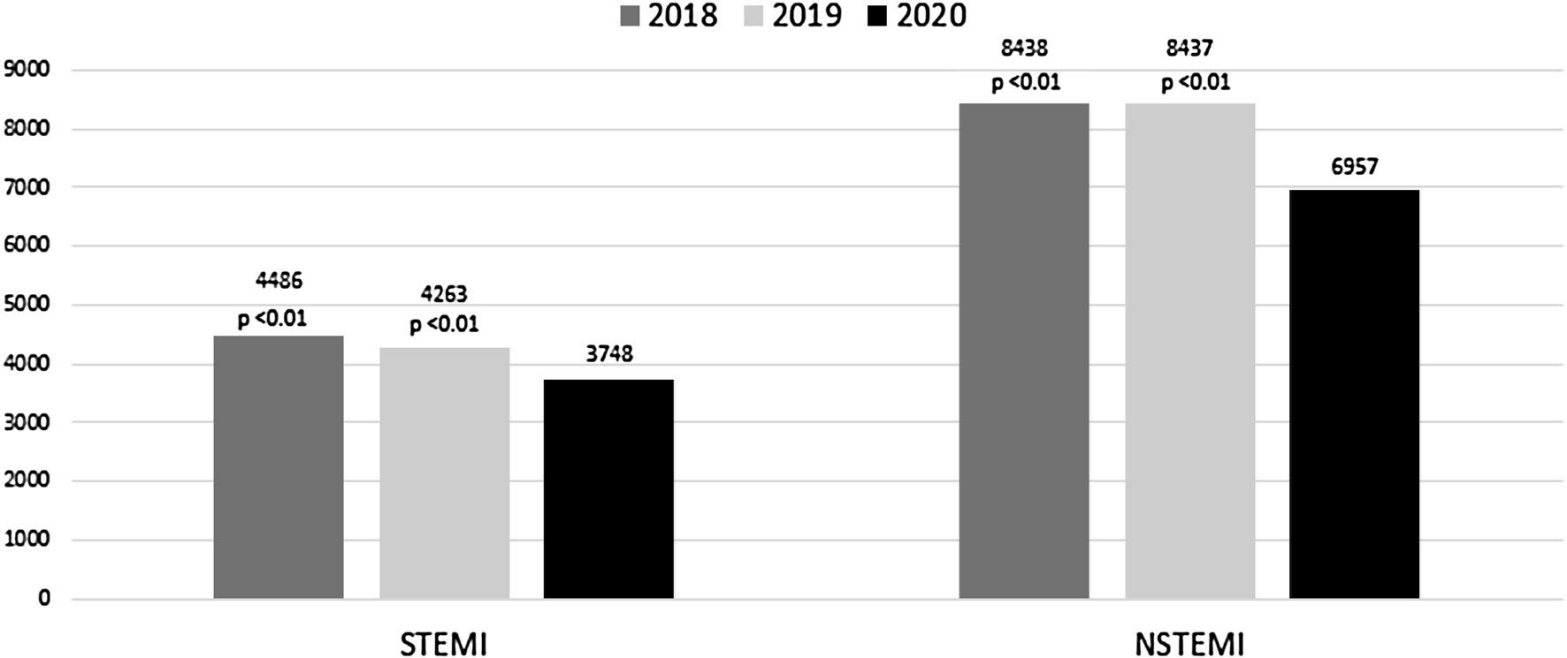
Comparison of admissions for STEMI and NSTEMI in the years 2018, 2019 and 2020

**Fig. 3:**
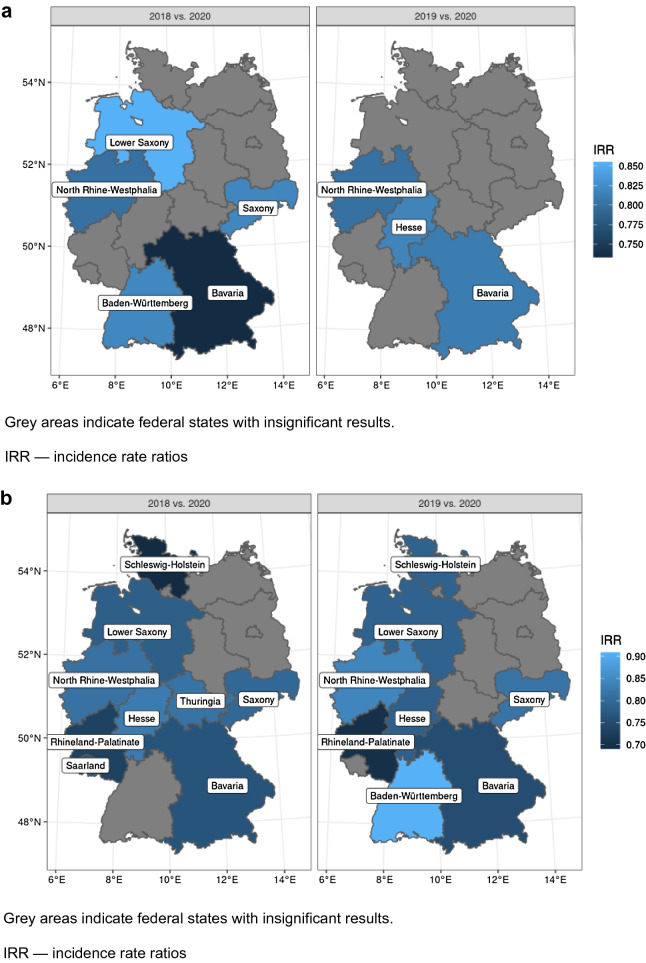

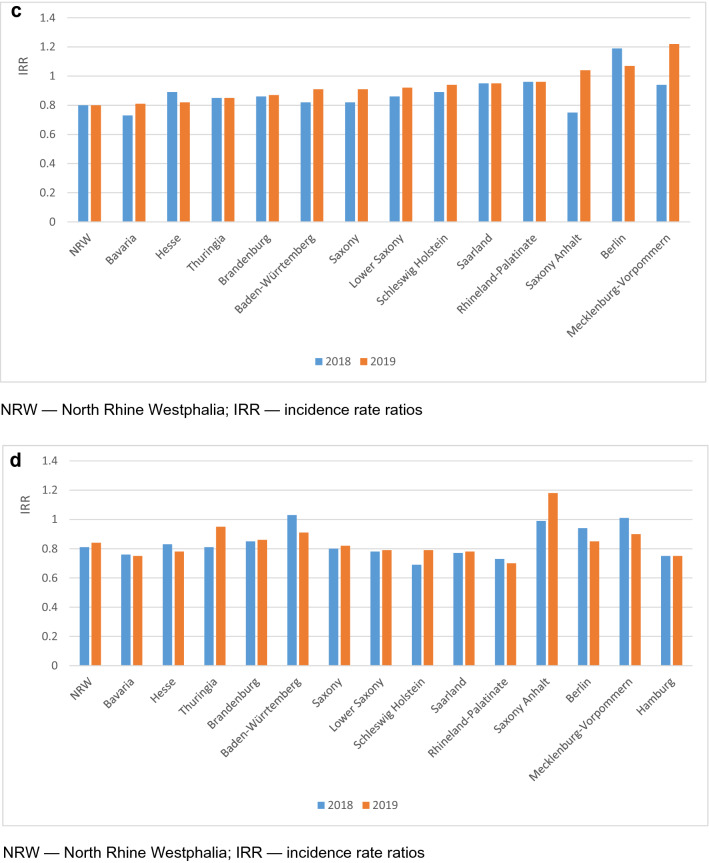
**a** Admission rate analyses for STEMI in German federal states in the years 2018, 2019 and 2020. **b** Admission rate analyses for NSTEMI in German Federal states in the years 2018, 2019, 2020. **c** Comparison of hospital monthly admissions for STEMI in different German federal states in the years 2018, 2019, 2020. **d** Comparison of hospital monthly admissions for NSTEMI in different German federal states in the years 2018, 2019, 2020. Grey areas indicate federal states with insignificant results. IRR—incidence rate ratios; NRW—North Rhine Westphalia

The number of patients admitted in the 3 years 2018, 2019 and 2020 are given in Fig. 2 (STEMI: 3,748 in 2020, 4,263 in 2019 and 4,486 in 2018; *p* < 0.01; NSTEMI: 6957 in 2020, 8437 in 2019 and 8438 in 2018; *p* < 0.01). The incidence rate ratio for STEMI in 2020 was 0.88 (95% CI 0.84–0.92) versus 2019 and 0.84 (95% CI 0.80–0.87) versus 2018, while the corresponding incidence ratios for NSTEMI were 0.82 (95% CI 0.80–0.85) versus 2019 and 0.82 (95% CI 0.80–0.85) versus 2018. In Fig. 3a and b, the differences between the Federal states of Germany in the decline of admissions are depicted. Interestingly, there was no significant interaction between the number of SARS-CoV-2 infections per 100.000 inhabitants in the respective Federal states and the year of admission.

The baseline characteristics and concomitant diseases for patients with STEMI and NSTEMI are given in Table 1. Age and gender distribution did not differ between the years, while a lower Elixhauser comorbidity index was observed in 2020 in patients with NSTEMI but not with STEMI.

**Table 1:** Baseline characteristics

Year	STEMI				NSTEMI			
	2020	2019	2018	*p*-value	2020	2019	2018	*p*-value
Age < 70 years	62.3% (2273)	63.0% (2645)	60.6% (2670)	< 0.01	44.4% (3031)	43.8% (3658)	43.4% (3590)	0.64
Age > 80 years	17.1% (624)	17.1% (720)	18% (792)	0.49	29.3% (2004)	28.6% (2387)	27.3% (2261)	0.02
Male sex	70.2% (2610)	72.1% (3062)	72.1% (3211)	0.71	67.2% (4646)	67.5% (5683)	67.5% (5647)	0.09
Elixhauser Comorbidity index	7.8 ± 9.9	7.6 ± 9.8	8.0 ± 10.0	0.14	8.0 ± 9.8	8.3 ± 10.0	8.7 ± 10.1	0.01
Hypertension	16.6% (596)	17.7% (722)	18.0% (773)	0.27	21.8% (1444)	21.9% (1772)	24.1% (1942)	0.01
Diabetes	5.5% (197)	5.8% (236)	5.1% (218)	0.36	10.2% (674)	10.5% (854)	9.7% (780)	0.17
Renal failure	16.9% (606)	16.2% (664)	15.8% (681)	0.43	26.3% (1735)	27.7% (2243)	27.3% (2202)	0.13
Congestive heart failure	44.0% (1577)	43.8% (1789)	44.5% (1912)	0.81	43.6% (2884)	45.5% (3685)	45.9% (3707)	0.01
Valvular disease	12.0% (431)	11.7% (477)	12.6% (540)	0.45	18.8% (1240)	18.8% (1518)	18.8% (1520)	0.99
Chronic pulmonary disease	5.4% (195)	5.8% (239)	6.6% (282)	0.10	9.1% (602)	9.8% (793)	9.8% (788)	0.29
Metastatic cancer	0.3% (9)	0.3% (13)	0.2% (8)	0.48	0.6% (38)	0.5% (43)	0.3% (23)	0.02

STEMI—ST-elevation myocardial infarction; NSTEMI—non-ST-elevation myocardial infarction; CI—confidence interval

### In-hospital care

**Fig. 4:**
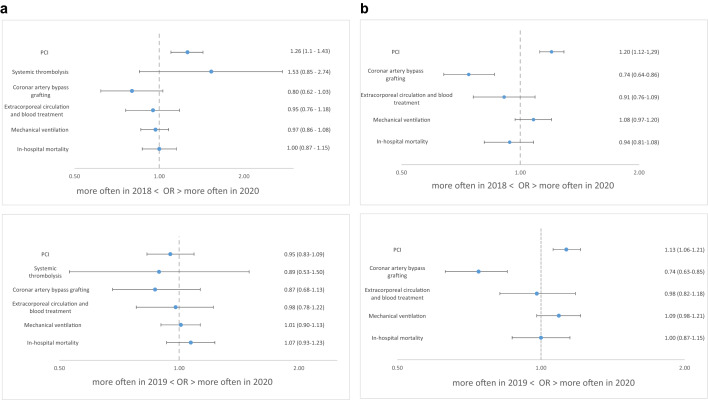
**a** Treatments and in-hospital outcomes for STEMI in the years 2018, 2019, 2020. **b** Treatments and in-hospital outcomes for NSTEMI in the years 2018, 2019, 2020

In-hospital care is shown in Table 2. PCI was performed more often in 2020 than in 2019 (odds ratio 1.13, 95% CI 1.06–1.21) and 2018 (odds ratio 1.20, 95% CI 1.12–1.29) in NSTEMI and more often than in 2018 (odds ratio 1.26, 95% CI 1.10–1.43) in STEMI, while there was no difference in the rate of coronary artery bypass graft (CABG) procedures (Fig. 4a, b).

**Table 2:** In-hospital procedures

Year	STEMI				NSTEMI			
	2020	2019	2018	*p*-value	2020	2019	2018	*p*-value
Fibrinolysis	0.7% (27)	0.8% (33)	0.5% (21)	0.16	–	–	–	
PCI	86.1% (3270)	85.9% (3734)	82.6% (3762)	< 0.01	56.8% (4017)	54.2% (4667)	52.0% (4441)	< 0.01
CABG	3.9% (149)	4.3% (186)	4.6% (209)	0.09	6.5% (460)	8.4% (723)	8.7% (743)	< 0.01
Extracorporeal circulation and blood treatment	4.0% (151)	4.1% (177)	4.2% (191)	0.63	3.0% (210)	3.2% (272)	3.5% (295)	0.31
Mechanical ventilation	17.5% (663)	17.4% (757)	18.2% (828)	0.56	11.1% (787)	10.4% (896)	10.9% (928)	0.10
Intensive care treatment	60.3% (2290)	59.0% (2568)	62.8% (2863)	< 0.01	36.8% (2603)	37.8% (3258)	41.2% (3518)	< 0.01

STEMI—ST-elevation myocardial infarction; NSTEMI—non-ST-elevation myocardial infarction; PCI—percutaneous coronary intervention; CABG—coronary artery bypass grafting

Intensive care treatment was reported for STEMI in 60.3% in 2020, 59.0% in 2019 and 62.8% in 2018 (*p* < 0.01 versus 2020). The rates for NSTEMI were 36.8% in 2020, 37.8% in 2019 and 41.2% in 2018 (*p* < 0.01 versus 2020), respectively.

Length of stay was shorter in 2020 compared to 2019 and 2018 (mean 6.7 days versus 7.7 days versus 7.6 days; both *p* < 0.01) in STEMI and NSTEMI (mean 6.1 days versus 7.4 days and 7.7 days; both *p* < 0.01), respectively.

### In-hospital mortality

In-hospital mortality rates (Fig. 4a, b) were similar both for STEMI (2020: 12.5%, 2019: 11.8%, 2018: 12.5%) and NSTEMI (2020: 6.6%, 2019: 6.7%, 2018: 7.2%), respectively.

## Discussion

The COVID-19 Collateral Damage analysis is the largest study on the impact of the first wave of COVID-19 on care of patients with acute myocardial infarction in Germany. The results can be summarized as follows: (1) the first wave decreased STEMI and NSTEMI admissions in Germany in comparison to 2018 and 2019; (2) these effects on admissions were more pronounced in NSTEMI; (3) in-hospital mortality was unaffected for both STEMI and NSTEMI; (4) guideline-directed therapy including PCI was even higher in comparison to previous years; and (5) incidence rates of COVID-19 infections in different regions did not correlate with differences in regional STEMI and NSTEMI admission rate reductions.

### Impact on ACS admissions

The impact of COVID-19 on the incidence, acute care and mortality of acute myocardial infarction is still controversial. It has been shown that COVID-19 causes acute heart damage leading to worsening of pre-existing systolic dysfunction, cardiogenic shock, tachyarrhythmias, or ACS [26]. Several studies suggest a possible inflammatory pathophysiological mechanisms that can trigger plaque disruption and create a prothrombotic milieu [27, 28]. In addition, stressful events usually trigger more ACS. Therefore, an increase in the number of patients with ACS during the COVID-19 pandemic was to be expected. However, initial reports from small registries have shown remarkable reductions in the number of patients admitted with ACS [10–12, 14]. It has been speculated that during the first lockdown patients avoided to seek hospital care because of the fear of COVID-19 infections or to avoid burdening an already overwhelmed health service. Alternative explanations for the reduced number of ACS admissions include less stress because of the increase in home-office work and reduced social contacts and less air pollution because of decreased car and air traffic [29].

The current results are very much in line with the published data from other countries and regions. Thus, it can be stated that the first COVID-19 wave has been associated with a decrease of ACS admissions.

### Impact on ACS mortality

COVID-19 may have increased cardiovascular mortality both through direct and indirect impacts on the incidence and management of acute heart disease. These behaviors can lead to increased morbidity and mortality, especially in patients with STEMI, in whom a longer time delay has a significant negative impact on myocardial salvage and maintenance of left ventricular function [30, 31].

The absolute impact of the COVID-19 pandemic on mortality of patients with ACS must be divided into two populations, the first one dying at home or before hospital admission and the second one admitted to the hospital and dying after admission. Here, we can only report the results for the second group and we did not find any increase in in-hospital mortality compared to the previous two years. In the Spanish ISACS-STEMI COVID-19 registry, where a total of 6609 STEMI patients were analyzed and compared with 2019, there was a significant reduction in the number of primary PCI procedures during the pandemic (in 2020) compared to 2019, especially for patients with arterial hypertension [32]. In addition, a higher mortality rate was observed during the COVID-19 pandemic first wave. There are similar reports from other countries such as Italy and the United Kingdom [33, 34]. The reasons for the reported higher mortalities might be different according to region, health-care systems and in some reports due to chance because of small sample size. Based on the overall COVID-19 infection rate some regions allocated all resources to the care of COVID-19 patients, leading to a lower use of primary PCI and thus higher mortality.

These observations on mortality could not be confirmed by our study, with a representative sample size from German hospitals. In these hospitals revascularization procedures and in-hospital mortality did not differ between 2018, 2019 and 2020, both in NSTEMI and STEMI patients. This might be due to the fact that German hospitals were still able to adequately care for ACS patients in 2020 and were not reaching their limit of capacities because of the care for COVID-19 patients.

However, it might well be that overall mortality of ACS patients has been increased due to a higher number of patients dying at home. This hypothesis is supported by the findings from northern Italy with an increase of the number of out-of-hospital cardiac arrests during the first wave. Therefore, it seems that ACS patients stayed at home too long and did not seek medical care because of the fear to get infected in the hospitals. These findings are in line with an analysis from central Germany in which an 8.5% increase in cardiovascular mortality has been reported during the first wave, due to an increase of in-hospital and out-of-hospital mortality. We could not determine the effect of COVID-19 on the overall cardiovascular mortality in Germany, but the increased total mortality for the years 2020 and 2021 (with the highest mortality rate of 1.023.723 cases in 2021 since 1946) as reported in the data of the “Statistisches Bundesamt” support the likelihood of an increase of out-of-hospital mortality in ACS patients [35]. Other factors like delay in treatment for cardiovascular disease such as heart failure, aortic stenosis, or also delay in treatment for cancer may have contributed to the excess in mortality in these years. Future analyses will have to explore the underlying causes.

### Limitations

Our data derive from the analysis of routine data, which primarily were not collected for research purposes. Therefore, coding errors or missing coding could have an impact on the results. Unfortunately, these data also lacks more precise information, such as ischemia time and door-to-balloon time.

## Conclusion

This study is the first in which the relevant data on patients with acute myocardial infarction were collected and analyzed from nearly all the Federal states of Germany. During the first wave of the COVID-19 pandemic, significant reductions in patient admissions have been observed both for STEMI and NSTEMI, while in-hospital treatment and in-hospital outcomes were not affected. Future studies will have to explore the underlying reasons for the excess in overall mortality in Germany in 2020 and 2021.

## Data Availability

Available on reasonable request.
